# Bullous pemphigoid treated with baricitinib as steroid-sparing therapy for a patient with uncontrolled diabetes

**DOI:** 10.1016/j.jdcr.2024.12.017

**Published:** 2024-12-30

**Authors:** Ji Won Heo, Youngkyoung Lim

**Affiliations:** a Seoul National University College of Medicine, Seoul, South Korea; b Department of Dermatology, Veterans Health Service Medical Center, Seoul, South Korea

**Keywords:** baricitinib, bullous pemphigoid, diabetes, JAK inhibitor, steroid-sparing therapy

## Introduction

Bullous pemphigoid (BP) is an autoimmune subepidermal blistering disease characterized by tense bullous lesions and pruritus, primarily affecting older adults.^1^ Autoantibodies like BP180 and BP230 are key in BP’s pathogenesis, targeting the basement membrane and causing separation at the dermal–epidermal junction.^1^^,^^2^ Patients with moderate to severe BP often receive high-dose corticosteroids with tapering. However, long-term use of high doses requires careful consideration due to potential adverse effects on glycemic control. This concern is particularly relevant in BP patients, given the relapsing nature of the disease and the high prevalence of diabetes among older adults.^3^ We present a case of an elderly BP patient with uncontrolled diabetes who achieved remission with baricitinib without metabolic adverse events.

## Case report

**Fig 1:**
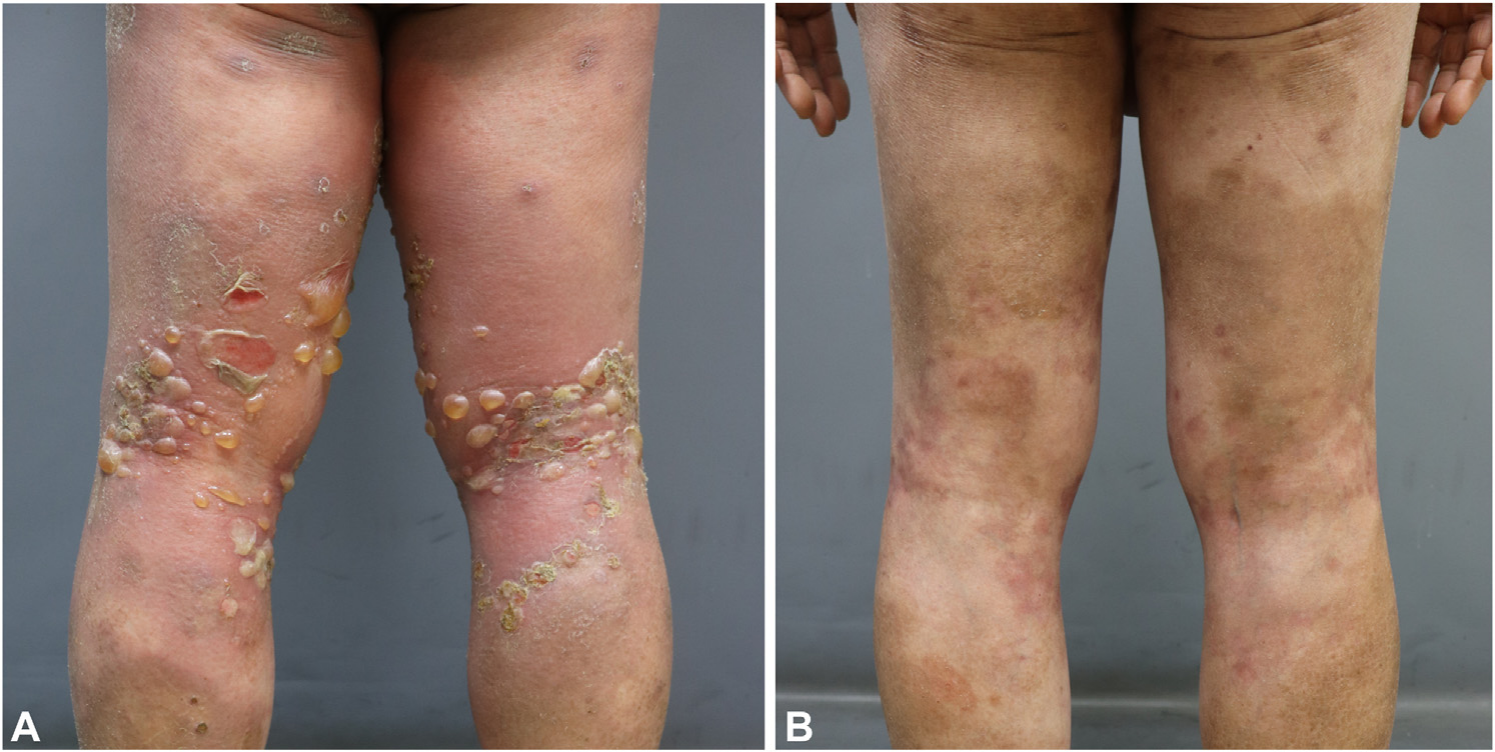
Clinical images of a male patient with bullous pemphigoid before and after treatment with baricitinib. **A,** Bullous pemphigoid lesions on both legs with erythematous patches and tense bullae with crusted erosions before treatment. **B,** Bullous pemphigoid mostly resolves with residual postinflammatory hyperpigmentation but without new blisters after treatment.

A 75-year-old male patient presented with multiple blisters on the trunk and extremities associated with severe pruritus. The patient had no history of medication that could have caused lesion development. A physical examination revealed painless erythematous patches and multiple tense bullae with crusted erosions after rupture (Fig 1, *A*). Therefore, a biopsy of the early blisters on the abdomen was performed, revealing subepidermal bullae with eosinophils in the bullous cavity and interstitium of the dermis. Coarse granular pseudolinear staining for immunoglobulin G and complement 3 in the dermal–epidermal junction was observed with direct immunofluorescence. Among the differential diagnoses of subepidermal immunobullous diseases, a diagnosis of BP was made with comprehensive assessment of direct immunofluorescence pattern (linear deposition of immunoglobulin G and complement 3), patient age, and characteristics of lesions (absence of atrophic scars, predominance in trunk and extremities without mucosal involvement).

The patient had diabetes and hypertension, for which he received triple therapy (metformin, pioglitazone, and empagliflozin) and amlodipine, respectively. The initial laboratory results revealed hypereosinophilia (27%, absolute eosinophil count, 5013 cells/uL), marked hyperglycemia (random glucose, 328 mg/dL; glycated hemoglobin, 9.1%), and reduced renal function (estimated glomerular filtration rate, 38 mL/min/1.73 m2). Oral steroids (methylprednisolone 24 mg) were initially prescribed as the first-line treatment, combined with antihistamines, antibiotics, and topical steroids.

After consulting endocrinology for glucose monitoring, high-dose steroids were started during admission. However, due to severely uncontrolled glucose level, oral steroids were tapered over 2 weeks and completely discontinued. We considered several alternatives including tetracycline antibiotics, immunosuppressants, and novel targeted treatments such as omalizumab, dupilumab, and Janus kinase (JAK) inhibitors. Given the patient’s disease severity, immunosuppressants were preferred over doxycycline, which is effective in mild cases. Among immunosuppressants, cyclosporine was considered as a steroid-sparing medication but was deferred because of the patient’s chronic kidney disease. Considering the patient’s age and comorbidities, novel targeted agents, dupilumab and JAK inhibitors, with less hepatic and renal toxicity, were considered. Baricitinib (4 mg/day) was initiated considering patient preference for oral medication. Screening for infections, including tuberculosis, revealed negative results.

Clinical improvement in pruritus and skin lesions was noted 1 week after initiating baricitinib treatment. Additionally, the treatment was maintained without the emergence of new lesions. Complete remission, with minimal postinflammatory hyperpigmentation, was achieved 10 weeks after starting the treatment (Fig 1, *B*). After reaching the maintenance phase, baricitinib was gradually tapered to reduce potential adverse effects while also minimizing the risk of flares due to the dose reduction. With close monitoring, the dosage was reduced from 4 mg daily for 4 months to 4 mg every other day for 3 months and is currently maintained at 4 mg every 3 days, which has been ongoing for 7 months without any flares.

Overall, the patient exhibited excellent tolerance to treatment for 14 months of follow-up with improved glycemic control (glycated hemoglobin, 6.4%; glucose, 137 mg/dL) without side effects on lipid profile (triglyceride, 102 mg/dL; low-density lipoprotein cholesterol, 72 mg/dL (normal <130); high-density lipoprotein cholesterol, 58 mg/dL) or renal function (creatinine, 1.9 mg/dL). Relapse was not observed during follow-up.

## Discussion

The JAK/signal transducers and activators of transcription pathway has been actively investigated in inflammatory skin diseases due to its central role in immune regulation and interaction with signaling molecules.^4^ Baricitinib is a JAK 1/2 inhibitor that also inhibits cytokines involved in the differentiation of T helper 17 (Th17) cells.^4^ Although the exact mechanisms are still under investigation, several case reports have described clinical improvements in BP with JAK inhibitors (Table I).^5^, ^6^, ^7^, ^8^, ^9^

**Table I:** Patient profile and treatment regimen comprising Janus kinase inhibitors for bullous pemphigoid

Patient	Sex	Age (yr)	Treatment	Dosage	Time to CI	Time to complete remission	Trial before JAK inhibitor treatment	Medical history	Reference
1	M	83	Baricitinib	4 mg/day orally, halved after 12 wk	12 wk	24 wk	None	Plaque psoriasis, stage 3 HTN	Xiao et al^5^
2	M	33	Tofacitinib	5 mg twice a day, halved after 24 wk	24 wk	24 wk	Avelox for psoriasis, systemic GC + cyclosporine	Psoriasis, HTN attributable to cyclosporine	Li et al^6^
3	F	65	Tofacitinib	10 mg twice a day	1 wk	12 wk	Prednisone + doxycycline + niacinamide	Hypothyroidism, obesity, OA, seronegative SpA on adalimumab, sulfasalazine	Youssef et al^7^
4	M	76	Tofacitinib	10 mg twice a day	3 wk	3wk	Systemic GC + mycophenolate + dupilumab	Steroid-induced atrial fibrillation	Youssef et al^7^
5	M	67	Tofacitinib	5 mg twice a day	6 wk	14 wk	Systemic GC + minocycline + nicotinamide		Fan et al^8^
6	M	10			4 wk	12 wk	Systemic GC + mycophenolate mofetil, topical GC + dupilumab	Diabetes mellitus, osteoporosis	
7	M	93			6 wk	16 wk	Topical GC + minocycline, dupilumab	HTN	
8	F	49			2 wk	8 wk	Systemic GC + minocycline + nicotinamide, topical GC + dupilumab	Cataract	
9	M	75			4 wk	12 wk	Systemic GC + minocycline		
10	M	86			8 wk	18 wk	Systemic GC + minocycline, systemic GC + azathioprine		
11	F	72			10 wk	12 wk	Systemic GC + cyclosporine, azathioprine		
12	F	81	Upadacitinib	15 mg orally	8 wk	8 wk	Prednisone 50 mg	HTN, dyslipidemia, OA, endometriosis	Nash and Kirchhof^9^
13	M	64	Baricitinib	4 mg orally	1 wk	8 wk	Methylprednisolone 4 mg + doxycycline 200 mg, systemic antihistamines, topical steroids	HTN, type 2 diabetes mellitus	Jun et al^3^

*BP*, Bullous pemphigoid; *CI*, clinical improvement; *GC*, glucocorticoid; *HTN*, hypertension; *JAK*, Janus kinase; *OA*, osteoarthritis; *SpA*, ankylosing spondylitis.

Type 2 inflammation has been implicated in the pathogenesis of BP, playing a role in autoantibody production through B cell stimulation and eosinophil chemoattraction.^1^^,^^4^ This is supported by clinical responses observed with dupilumab, which targets type 2 inflammatory cytokines like interleukin (IL)-4 and IL-13. However, varied manifestations, prognoses, and partial responses to these targeted agents in BP require further exploration.

Le Jan et al^2^ revealed increased IL-17 levels in BP blister fluid, suggesting the influence of Th17 responses on BP manifestations. Th17 cells produce IL-17, activating neutrophils that release neutrophil elastase which may contribute to the degradation of the dermal-epidermal junction. This mechanism helps explain the propagation of subepidermal blistering initially triggered by basement membrane-targeting autoantibodies.^10^ Therefore, we hypothesized that JAK inhibitors, by blocking Th17 differentiation, could cumulatively reduce blister formation and propagation, as evidenced by significant clinical improvement in blisters observed in this case. This offers new insights into targeted treatment of BP beyond just Th2 inflammation.

Baricitinib, as a novel immunotherapeutic agent, can serve as an alternative to high-dose corticosteroids when there are concerns about metabolic adverse effects. This may be particularly beneficial for older patients, who often have a high prevalence of diabetes, hypertension, and osteoporosis. However, given the limited reports on JAK inhibitors and potential adverse events—such as thrombosis, malignancy, and serious infections—discussions regarding the risks and benefits of treatment should involve the patient, and careful monitoring is warranted. Therefore, we discussed treatment options with the patient and decided to carefully taper to an optimal dose that maintains disease control while reducing the risk of adverse events.

In summary, we encountered an older patient who achieved complete remission of BP using baricitinib without worsening of existing comorbidities. This case not only broadens the scope of potential drug targets for BP but also highlights baricitinib as a viable treatment option for older patients who cannot tolerate corticosteroids due to systemic side effects.

## Conflicts of interest

None disclosed.
